# Kaumātua Mana Motuhake: peer education intervention to help Māori elders during later-stage life transitions

**DOI:** 10.1186/s12877-020-01590-z

**Published:** 2020-05-29

**Authors:** John G. Oetzel, Michael P. Cameron, Mary L. Simpson, Rangimahora Reddy, Sophie Nock, Hineitimoana Greensill, Pare Meha, Kirsten Johnston, Truely Harding, Pita Shelford, Linda Tuhiwai Smith, Brendan Hokowhitu

**Affiliations:** 1 University of Waikato, Private Bag 3105, Hamilton, 3240 New Zealand; 2 Rauawaawa Kaumātua Charitable Trust, 50 Colombo St., Hamilton, 3204 New Zealand

**Keywords:** Kaupapa Māori, Tuakana-teina, Peer education, Positive ageing, Community-based participatory research, Mana motuhake

## Abstract

**Background:**

Aotearoa/New Zealand has a population that is ageing and there are challenges to health and social outcomes related to related to key life transitions (e.g., retirement, change in health conditions, loss of spouse). Further, there are significant inequities between Māori (Indigenous people) and non-Māori in ageing outcomes. The purpose of this study was to test the impacts and cost effectiveness of a tuakana/teina (peer education) intervention on kaumātua (elders) receiving the intervention. This study was framed by a strengths-based approach based on the key cultural concept of mana motuhake (autonomy and self-actualisation).

**Methods:**

This study was grounded in principles of Kaupapa Māori and community-based participatory research to bring together a diverse group of stakeholders to co-develop and co-evaluate the intervention. The intervention had tuakana (peer educators) having conversations with up to six teina (recipients) and providing information related to health and social services. The research design was a pre- and post-test, clustered staggered design. Participants completed a baseline assessment of health and mana motuhake measures consistent with Māori worldviews along with two follow-up assessments (one after the first intervention group completed its activities and a second after the second intervention group completed its activities). Additionally, five focus groups and open-ended questions on the assessments were used to provide qualitative evaluation.

**Findings:**

A total of 180 kaumātua were recruited to the intervention with 121 completing it. The analysis revealed improvements over time in the expected direction on most of the variables. However, only three of the variables had statistically significant intervention effects: received support, tribal identity, and trouble paying bills. Qualitative results supported impacts of the intervention on mana motuhake, social connectedness, and tangible/information support related to services. Cost-effectiveness analysis showed that the intervention is cost effective, with a cost per QALY of less than the conventional threshold of three times gross domestic product per capita.

**Conclusions:**

The findings support the relevancy and importance of kaumātua knowledge to create a strengths-based approach to improve health and social outcomes. This study demonstrates that a contextually based and culturally safe age-friendly environments can facilitate engagement and participation by kaumātua for kaumātua.

**Trial registry:**

Australia New Zealand Clinical Trial Registry (ACTRN12617001396314); Date Registered: 3 October 2017 (retrospectively registered); https://www.anzctr.org.au/Trial/Registration/TrialReview.aspx?id=373733&isClinicalTrial=False

## Māori words

Aotearoa New Zealand.

Aroha Love, compassion, empathy.

Hauora Health.

Hinengaro Mind, thought, intellect.

Kaumātua Elders.

Kaupapa Māori Research by Māori for Māori.

Kete Basket.

Mana motuhake Autonomy, identity and self-actualisation.

Manaakitanga Enhancing self-esteem (in this study).

Māori Indigenous people of New Zealand.

Marae Community meeting space.

Mātāpono Principles.

Mātauranga Indigenous knowledge.

Pono Truth, integrity.

Rangatiratanga Taking action (in this study).

Tautoko Advocacy, support.

Tautokotanga Strengthen access to information (in this study).

Teina Peer education recipient.

Tika Correctness, justice, fairness.

Tikanga Customs and protocols.

Tuakana Peer educator.

Tuakana-teina Older sibling/younger sibling.

Waiora Wellbeing.

Wairua Spirit.

Wairuatanga Realising potential (in this study).

Whakawhanaungatanga Making social connections.

Whānau Extended family.

Whanaunga (tanga) Social wellbeing and connections.

## Background

The population in Aotearoa/New Zealand (hereafter referred to as ‘Aotearoa’), as well as with much of the world, is ageing and with this comes surges in injuries, health problems, and healthcare costs and other burdens on the socio-economic system [1]. In addition, significant inequities exist between Māori (Indigenous people of Aotearoa) and non-Māori in terms of ageing and health outcomes [2–4], which have cultural, social, individual and economic costs [2–4].

People encounter significant transition points as they age, such as retirement, loss of spouse, loss of independent living and changing health conditions [1, 5, 6]. Being able to traverse these transitions well involves handling service systems and socio-economic and emotional aspects, whilst having to rely on others more [7–11]. Kaumātua (elders) who are unable to cope with these transitions may face many deleterious consequences such as social isolation, lower quality of life, and reduced health outcomes [12, 13]. The impact of these transitions for kaumātua are considerable given the context of significant health and social inequities.

This study used a strengths-based approach that highlights the potential of kaumātua (elders) to be solutions to their own challenges building on the strength of their status or mana within Māori culture. Māori culture reveres its elders from the way that Māori whānau (extended family) honour them to the way that tikanga (protocols and customs) on the marae (community meeting space) and in various community settings highlight their importance [14, 15]. This strengths-based approach is what we call kaumātua mana motuhake; mana motuhake is a concept that foregrounds independence and autonomy to achieve actualisation and so that kaumātua can have high quality of life for self and others [16]. Mana motuhake emphasises collective determination and upholding tino rangatiratanga (independence) and mana (status and prestige as viewed by self and others) in defining problems and identifying solutions. In this manner, kaumātua are viewed as key knowledge holders and leaders for the collective benefit of Māori communities. This approach is in contrast to prominent deficit models for health inequities focusing on weakness and dependency [2]. This study is part of the Ageing Well National Science Challenge in Aotearoa (https://www.ageingwellchallenge.co.nz/), which emphasizes positive ageing as part of the government’s strategic approach to science investment.

Specifically, the intervention for this study was a tuakana-teina (literally, older sibling-younger sibling) peer-educator model where kaumātua work with other kaumātua [16]. The research literature on peer education builds on theories such as the theory of reasoned action [17], diffusion of innovation theory [18], and social learning theory [19] to demonstrate that peer support/education is effective for improving numerous health, social, and economic conditions [20–23]. Peer education includes a variety of activities delivered by non-professionals to people who are of similar characteristics (e.g., age, health, culture) and experiencing a health or social need [24]. Peer education/support creates new social relationships/networks, often constructed by social and health service providers, and these relationships/networks may include self-help groups [24]. Thus, peer education is distinct from family, organisational, and community support networks where there are pre-existing relationships.

Peer education has been used to assist people in managing various life-transitions, including chronic and acute social and health issues [24]. They are primarily employed within younger populations, but elder peer educators have recently featured in palliative care [25–27], raising awareness of health [28, 29], successful ageing [30], self-management of chronic conditions [31], and physical activity and fall-prevention in older age [32–35]. These efforts have demonstrated positive effects of peer education on the recipients.

The current study included two broad outcomes of peer education: hauora (holistic health) and mana motuhake. Māori models of health often include at least four elements of hauora or waiora (health and wellbeing): tinana (physical well-being), hinengaro (mental and emotional well-being), wairua (spiritual well-being), and whanaunga (social well-being). This holistic perspective of health reflects Māori views of the relationship of people to all aspects in the world [36]. Mana motuhake is indicated by such elements as autonomy, economic wellbeing, and life satisfaction [16].

In addition, the peer education model has culturally appropriate features. Tuakana-teina is a Māori customary concept [15] emphasising the relationship between an elder and younger sibling (or cousin) of the same biological sex. However, tuakana-teina has been applied to situations based on training and experiences; this is because the concept is based on reciprocity and responsibility and can be pedagogically applied in peer-education mentor/mentee settings [37]. The tuakana-teina peer-education model in the present research is based on a number of cultural values including pono (truth, integrity, faithfulness), aroha (love, compassion, mercy, empathy) and tika (correctness, justice, fairness) [15, 38]. Further, the peer-education model develops age-friendly social environments that value knowledge and cultural concepts such as whakawhanaungatanga (making social connections), tautoko (advocacy, support) and mātauranga (Indigenous knowledge) [14, 15, 37].

This project sought to answer a single broad research question: What are the outcomes of a ‘tuakana-teina’ peer-educator model, where kaumātua work with other kaumātua in relation to wellness, social integration/connectedness, engagement, life-enhancement, independence, and, in particular, significant life-transitions? This manuscript addresses two specific aims: a) To determine whether the peer education intervention enhanced the social and health outcomes (hauora and mana motuhake) for kaumātua receiving it; and b) to determine the cost-effectiveness of the intervention.

## Methods

The study protocol for this project was published elsewhere and full details of the project can be found there [39]. Thus, we only present in this paper a brief overview of the methodology, intervention, study design, measures, procedures, and data analysis.

### Methodology

The research was guided by Kaupapa Māori [40, 41] and adopted a community-based participatory research (CBPR) approach [42, 43]. Kaupapa Māori centralises Māori worldviews, mātauranga, and tikanga [44]. It is an approach that centres self-determination and local context by prioritizing Indigenous aspirations and history [41, 45], and is a for-Māori by-Māori approach to research [46, 47]. CBPR is a participatory research methodology that involves academic and community partners equitably in all phases of the research process [42].

The research team represents an ongoing partnership between Rauawaawa Kaumātua Charitable Trust (RKCT; an organisation that serves the health and social wellbeing needs of elders using a Māori philosophy) and researchers from the University of Waikato. We included two advisory groups to help support our methodology [41, 42]: (1) a Board Advisory Group comprised of the trustees of RKCT to ensure it was kaumātua-led; and (2) an Expert Advisory Group consisting of experts in social and health issues. The boards provided stewardship for the project regarding the intervention development, the content of the intervention, and all research methods.

### Intervention

We developed a “Tuakana-teina/peer education orientation programme” for life-transitions of kaumātua [48]. The programme was framed as an ‘orientation’ rather than a training programme, in order to reflect the participant driven nature of the project and also to emphasise that kaumātua have expertise and experience that they will draw on. It was developed over an eight-month participatory process and informed by prior research [27, 49, 50], but primarily driven by Māori tikanga and input by the advisory groups. It also included a pilot of the programme with revisions included after the pilot [48].

Orientation activities were delivered by members of the research team to the tuakana only. The orientation included the following elements: (a) Māori values and mātāpono (principles); (b) definitions of tuakana-teina-peer support-important skills/attributes; (c) four kinds of support (affirmational, cultural, emotional, and informational); (d) forms of Māori communication; (e) specific communication tools to support the tuakana in their conversations with teina; and (f) a resource kete (basket) consisting of health and social services that the tuakana could provide the teina. The final programme included four 4-h sessions over two weeks. The initial sessions focused on exploration of the research project and programme, with the later sessions focusing on the tuakana-teina relationship, skill development, and communication practice. A booster session was offered about four weeks after the initial session.

The teina were matched to a tuakana of the same sex by a research coordinator from RKCT who knew the participants well. Each teina was to have up to three conversations with his/her tuakana over a roughly 12–16 week period. Each tuakana was assigned five or six teina. The conversations were recorded in order to complete a fidelity check of tuakana communication.

### Study design, procedures, and sample

**Fig. 1:**
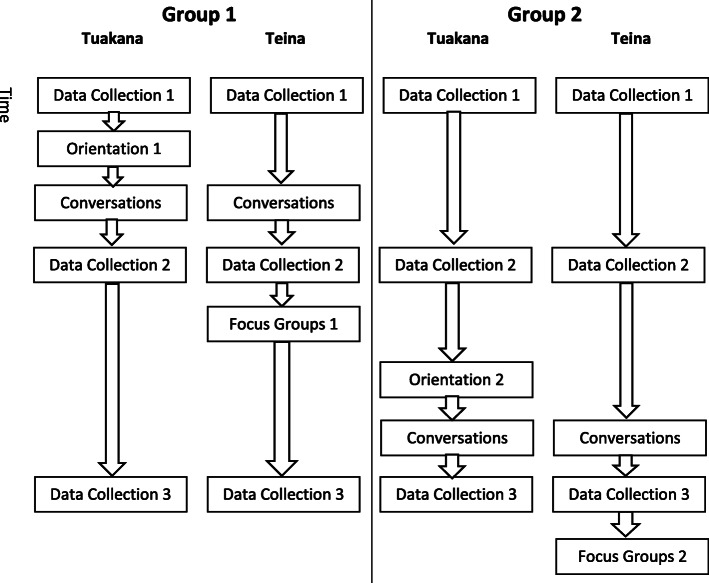
Flow diagram of research activities

The research design for the evaluation of the intervention was a pre- and post-test, clustered staggered design with two groups (G1 and G2). G1 participated in the orientation programme initially, while G2 participated in a subsequent orientation approximately eight months later. There were three data collections points for all participants with variations for the two groups: a) G1: pre-intervention, approximately four months post-intervention, and then four months after the second evaluation; b) G2: pre-intervention, approximately four months after initial survey (but before their orientation), and then four months post-intervention. Figure 1 displays a flow diagram of the research activities for the tuakana and teina in each group. Participants completed the survey via a paper-pencil form, and could have a support person present, who may have been the research administrator from RKCT. The staggered research design was chosen because it is consistent with Kaupapa Māori principles, in which withholding an intervention from participants is not ethical. This research design enabled a comparison of the two groups and is pragmatic for interventions in the health service sector [51]. The project was registered with the Australia New Zealand Clinical Trial Registry (ACTRN12617001396314). The research procedures were approved by the University of Waikato Human Research Ethics Committee through the Faculty of Māori and Indigenous Studies.

After the initial recruitment, there were a total of 13 tuakana in G1 and 16 in G2. By the completion of the project, there were 13 in each group. There were 485 teina eligible for recruitment and we randomly recruited teina participants from the list of eligible people. From this total, we had the following responses: unable to contact (*n* = 185, 38%), decline to participate (*n* = 120, 25%), and accept participation (*n* = 180, 37%). When we were unable to contact a person, a new person was randomly selected from the list; 60% of those contacted agreed to participate. We compared the age and biological sex between those who agreed to participate and those who did not and found no statistically significant differences, suggesting no selection bias in those agreeing to participate in terms of those variables.

We attempted to randomly assign the participants to G1 and G2 groups during the recruitment process. Some participants requested they be allocated to a different group than the group they were originally assigned to due to travel plans and other commitments and we decided to include them in the analysis given the methodology of the project. A total of 37 people (21%) were not randomly assigned. We compared the age and biological sex between those who were randomly assigned and those who were not and found no significant differences. From the original 180 teina participants recruited, a total of 121 participated in the intervention, having at least one conversation with a tuakana, for a retention rate of 67%. An additional 19 participants completed all three surveys, but were not assigned a tuakana (withdrawing from the intervention, but agreeing to participate in the surveys). The other 40 selected teina participants stopped participating in the study: 15 of these participants were assigned to a tuakana but did not participate in the intervention; 6 withdrew after first survey due to health of self or partner; 3 died during the study; 13 were unable to be contacted after second survey and no reason given; and 3 became tuakana for the G2 group. Three teina participants were elevated to tuakana status for the G2 group because six tuakana in G2 withdrew and there were not enough tuakana remaining for the selected teina. The third responses for those teina who were elevated to tuakana status are not included in the analysis.

### Measures

There were two core constructs for this study along with demographic items: hauora (health) and mana motuhake. Quantitative measures are detailed elsewhere [39] and were validated in a separate study [52]. Table 1 provides a list of the core constructs, number of items and Cronbach’s alpha calculated for this sample, where appropriate. In addition, an open-ended question was included on the survey asking participants to describe what they thought of the intervention and any recommendations they had for it.

**Table 1:** Constructs and Measures

Construct	Measures [source]	Number of Items	Cronbach’s Alpha
Hauora—tinana/hinengaro	Self-rated health [53, 54]	1	n/a
Hauora—tinana/hinengaro	Health-related quality of life (HRQOL) [55]	5	.88
Hauora-hinengaro	Likelihood of using services [39]	1	n/a
Hauora-wairua	Spirituality [56]	1	n/a
Hauora-whanaungatanga	Loneliness [1]	4	.57
Hauora-whanaungatanga	Tribal identity [13]	2	.90
Hauora-whanaungatanga	Importance of whānau [13]	1	n/a
Hauora-whanaungatanga	Knowledge of tikanga [13]	1	n/a
Hauora-whanaungatanga	Received social support [57]	2	.66
Hauora-whanaungatanga	Desired social support [57]	2	.60
Hauora-whanaungatanga	Perceived burden [58]	2	.78
Hauora-whanaungatanga	Perceived benefit [39]	2	.70
Mana motuhake	Economic wellbeing—trouble paying bills [56]	1	n/a
Mana motuhake	Economic wellbeing—trouble with housing [56]	1	n/a
Mana motuhake	Perceived autonomy [56, 59]	3	.80
Mana motuhake	Global life satisfaction [60]	1	n/a

We also conducted five focus groups with teina post-intervention (three in G1 and two in G2). The focus groups included a total of 22 participants (8 men and 14 women). Focus group questions explored participants’ experiences during the conversations with tuakana, the impact of the intervention on them and their whānau, and recommendations for the intervention in the future. The focus groups were administered by the research administrator from RKCT with support from a second organisation team member. The focus groups were audio-recorded and professionally transcribed.

### Data analysis

The first specific aim sought to determine whether the peer-educator model results in changes to hauora and mana motukahe. The analysis involved multilevel analysis of mixed models using SPSS 25.0 following procedures to isolate the effect of the intervention across different groups at different times using average treatment effect on the treated (ATT) [61, 62]. In addition, we included nesting of repeated measures within individual teina and teina within intervention group. We did not include non-intervention participants since many were not randomised to a group. We also included a significant demographic covariate that differed at baseline (i.e., age).

Thematic analysis was used to analyse the focus group and open-ended survey question data in order to support the quantitative results [63, 64]. Themes were identified using three criteria: repetition, recurrence, and forcefulness [65]. Analysis was completed by two researchers (one Māori and one non-Māori). The process involved several steps: a) reading of responses and identifying initial codes; b) identification of themes and exemplar quotes; and c) report back to advisory groups and participants for feedback. Feedback from other researchers and participants confirmed the analysis.

The second specific aim explored the cost effectiveness of the intervention using incremental cost effectiveness analysis (ICEA) [66, 67]. HRQOL was used as the outcome measure for the ICEA [68]. The incremental cost-effectiveness ratio (ICER) was converted to a cost-per-QALY (Quality-Adjusted Life Year). Following WHO practice [67], this cost-per-QALY was compared with GDP per capita as a threshold to determine whether the programme was cost-effective or not. As a robustness check, the cost-per-QALY was also compared with a measure of the value of a preventable life-year lost [69].

## Results

### Participants and participation

The average age of participants was 70.17 (SD = 7.48) and the sample was 65% female. Table 2 presents the demographic characteristics of the two intervention groups and the non-intervention participants at the pre-intervention/baseline period. The only significant difference was for age with the G1 group being younger than the other two groups. Additionally, there were no differences in the groups for any of the measures of the key constructs at baseline.

**Table 2:** Teina at Baseline

Variable	Category	G1	G2	Non-intervention participants
Age		68.20 (7.33)^a^	71.48 (7.20)^b^	71.46 (7.50)^b^
Biological Sex	Male	24	18	16
	Female	45	34	38
Unpaid utilities due to shortage of money	> 1	13	7	11
	1	6	8	3
	Not at all	49	36	40
Housing problems	Big problem	14	5	7
	Medium problem	9	3	5
	Small problem	15	15	13
	Not a problem	30	29	29
Others have financial control	Yes	9	8	10
	No	59	44	44
Relational Status	Married	22	20	14
	Other	47	32	40
Looking after mokopuna/grandchildren	Yes	14	12	16
	No	36	32	23
	Sometimes	18	8	15
Who else lives in house	Partner	20	19	16
	Children	15	15	9
	Mokopuna	13	12	16
	Flatmate	0	3	1
	Others	18	8	6
Know where to get services	Yes	50	42	47
	No	16	9	7
Like help to get services	Yes	18	13	23
	No	28	19	15
	Sometimes	22	18	14

Notes: Frequency or Mean (SD); Different superscripts indicates significant at *p* < .05

For the G1 group, there were a total of 71 teina who were assigned a tuakana. Of these teina, 61 had all three conversations with their tuakana, five had two conversations, three had one conversation, and two had no conversations. For the G2 group, there were a total of 65 teina who were assigned a tuakana. Of these teina, 28 had all three conversations with their tuakana, 12 had two conversations, 12 had one conversations, and 13 did not have any conversations. Table 3 displays the differences between the two groups on all this information. The differences in number of teina, conversations, missed conversations, and non-participants are statistically significant.

**Table 3:** Conversations and Teina in the Programme

Variable	Overall	G1	G2
Average Number of Teina	4.65 (SD = 1.35)	5.31 (SD = 0.75)	4.00 (SD = 1.53)
Average Number of Conversations	11.88 (SD = 5.28)	14.92 (SD = 2.78)	8.85 (SD = 5.51)
Number of Teina	121	69	52
Total Conversations	309	194	120
Number of missed conversations	49	13	36
Non-participants	15	2	13

The reasons for missed conversations were generally because people had changing obligations or health situations. This occurred more for the G2 group as they had to wait nearly nine months from initial commitment to the actual intervention. Additionally, the novelty and excitement of the programme had waned from the beginning of the project. These represent challenges when trying to develop and implement an intervention for older people with a staggered research design.

We completed the fidelity check by independently coding the conversations for communication features covered in the orientation programme (94% agreement within one-point among three coders; Scott’s pi = .88). These were organised around two categories: a) listening/respect and b) soliciting/sharing information. The average ratings were 2.36 (on a 3-point scale; SD = .40) for listening/respect and 2.73 (on a 3-point scale; SD = .38) for soliciting/sharing information, demonstrating relatively high levels of communication between the tuakana-teina pairs. There was no difference in these measures between the two intervention groups.

### Outcomes

#### Mixed model analysis

The first key aim was to have a positive impact on the social and health outcomes for teina. We completed an ATT analysis and all participants are included regardless of whether they received the full intervention (i.e. regardless of whether they had all of the planned conversations). Table 4 displays the means across the time for the participants and the ATT effect.

**Table 4:** Means of outcome variables across time for teina participating in the intervention and intervention effect

Variable	G1			G2			Intervention Effect (ATT)	SE
	Baseline, M (SD) ***n*** = 69	Time 2, M (SD) ***n*** = 67	Time 3, M (SD) ***n*** = 62	Baseline, M (SD) ***n*** = 52	Time 2, M (SD) ***n*** = 51	Time 3, M (SD) ***n*** = 46		
**Hauora Outcomes**								
Self-rated health (100-point)	59.12 (24.84)	60.30 (23.48)	61.29 (26.08)	59.23 (23.75)	57.25 (25.93)	65.65 (24.10)	4.47	3.69
HRQOL (100-point)	62.59 (21.46)	64.09 (19.57)	65.06 (23.22)	62.48 (21.53)	63.37 (20.86)	71.33 (21.74)	2.66	2.93
Spirituality (5-point)	4.13 (.98) *n* = 68	4.22 (.93)	4.15 (.97)	4.33 (.92)	4.12 (1.05)	4.00 (1.01)	0.17	0.15
Likelihood of seeking services (5-point)	2.67 (1.37) n = 67	2.85 (1.38)	2.89 (1.45)	2.96 (1.25)	3.27 (1.31)	3.24 (1.34)	−0.25	0.23
Loneliness (4-point)	1.94 (.47)	1.82 (.43)	1.73 (.47)	1.89 (.53)	1.86 (.53)	1.81 (.47)	0.05	0.07
Perceived support (4-point)	2.69 (.84)	2.96 (.84)	3.06 (.92)	3.00 (.86)	2.78 (.91)	3.16 (.74)	0.35*	0.14
Desired support (4-point)	1.85 (.66)	1.75 (.52)	1.74 (.69)	1.86 (.68)	1.70 (.58)	1.64 (.72)	0.03	0.11
Burden (4-point)	1.55 (.64)	1.56 (.68)	1.38 (.60)	1.67 (.74)	1.64 (.69)	1.59 (.77)	0.03	0.10
Benefit (4-point)	2.98 (.72)	3.07 (.76)	3.28 (.82)	3.20 (.80)	3.21 (.77)	3.23 (.93)	0.04	0.12
Tribal identity (5-point)	3.69 (1.06)	3.90 (1.15)	3.76 (1.10)	3.76 (1.16)	3.62 (1.18)	3.79 (1.02)	0.36*	0.17
Knowledge of tikanga (4-point)	3.00 (.93) n = 68	3.06 (.78)	3.19 (.81)	2.94 (.89)	3.08 (.87)	3.28 (.72)	0.04	0.12
**Mana Motuhake Outcomes**								
Autonomy (10-point)	8.83 (1.38)	8.86 (1.50)	8.89 (1.49)	8.96 (1.32)	8.95 (1.30)	9.28 (1.07)	0.11	0.23
Life satisfaction (10-point)	8.00 (1.97)	8.13 (1.68)	8.48 (2.09)	8.50 (1.93)	8.80 (1.52)	9.02 (1.60)	−0.27	0.30
Missed bill payments (3-point)	1.47 (.80)	1.33 (.70)	1.47 (.78)	1.43 (.73)	1.57 (.85)	1.47 (.81)	0.28*	0.12
House problems (4-point)	2.10 (1.19)	1.88 (1.10	1.74 (.99)	1.71 (1.02)	1.84 (1.08)	1.80 (1.04)	0.09	0.18

*p < .05

Table 4 illustrates improvements from baseline to final time point in most of the outcome variables and many of these were significant including HRQOL, desired support, loneliness, life satisfaction and knowledge of tikanga. However, only three of the variables had positive and statistically significant intervention effects: received support, tribal identity, and trouble paying bills. Inclusion of measures of the quality of communication and number of conversations as covariates did not significantly change the results and thus are not presented here.

#### Qualitative analysis

The focus groups and open-ended question on the survey also provided information about social and health outcomes. There were three themes: a) Mana motuhake—Realising potential; b) Whanaungatanga—Strengthened whānau and social connectedness; and c) Tautokotanga—Assistance with information and support (see Table 5 for description and example quotes; all names are pseudonyms).

**Table 5:** Summary of Themes and Subthemes for Impacts on Teina

Themes	Subthemes	Example Quotes (Pseudonym)
**Mana motuhake: Enhanced autonomy and independence**	Subtheme 1: **Wairuatanga—**Realising potential; self-efficacy/ confidence	I think this program is awesome. In way that it has woken or enlightened me. (Tau, 68, male)
		Ko te whaka kaha toru i to wairua taha i ringawa katoa. The continued strengthening of the spirit at all times. (Tua, 68, male)
		Makes you think about yourself and where you stand. (Kokako, 73, male)
	Subtheme 2: **Manaakitanga**—Enhancing self-esteem, identity, and wellbeing	It’s exchanging knowledge and understanding with a little bit of Māori in it too. It’s been a great difference to me. (Tāne, 78, male)
		I enjoyed this program because it gave me the courage to express myself and how I felt in my daily routines and it helped me. (Mihi, 66, female)
		Being Māori, the connections are more stable. A lot of the times we are geared to look good in front of the Pākehā. Kaumātua mana motuhake bridges that gap. (Mahanga, 69, male)
	Subtheme 3: **Rangatiratanga**—Taking action; making changes. Specific changes in self /actions taken	[The intervention] open [s] your eyes to more information. It triggered me how to deal with things in my life that weren’t pleasant. (Hei, 75, male)
		Helps you out of isolation, loneliness. Helps you participate again in things that move you forward. Takes away the shyness. Opens the door to stepping out. (Ara, 67, male)
		It’s good, socialising, getting some other kaumātua’s point of view, getting involved instead of being a recluse. (Ina, 75, female)
**Whanaungatanga: Strengthened whānau and social connectedness**	Strengthened whānau and social connectedness	I shared most of the things that I learnt, to my family, then it was my job to take them everything that I’d learnt, and also shared information with my neighbours. Told them what this programme had done for me as a person, and also filled them in, and sort of like questions, where things they could be helped with. (Hinemoa, female focus group participant)
		I would recommend others participate and I am enjoying writing my story for my mokos (grandchildren). (Arama, 68, male)
		The people I meet there and talk to. Everyone’s got a smile. You don’t need to get ignored. It’s you people that have boosted my life more you have a bit of a laugh. (Pahoro, 56, female)
**Tautokotanga: Strengthened access to information**	Strengthened knowledge about services or information that can make a difference to kaumātua	It helps you be not alone. The resources are here and the people are here. (Kara, 73, female)
		I found it was good, because I could learn, when I was speaking with [my tuakana], learn about the services that are provided for me, because there are services out there that you can tap into, that I didn’t know of. (Pare, female focus group participant)
		I can see the benefits and understand the opportunity of being able to make contacts with people that understand and can offer me assistance and advice. (Kiri, 70, female)

The first theme concerned the ways in which teina realised their mana motuhake—their autonomy and independence. Specifically, it focuses on teina reports of realising their potential, enhancing their self-efficacy, health, and wellbeing. Three subthemes capture these ideas: (i) Wairuatanga—Realising potential; (ii) Manaakitanga—Enhancing self-esteem, identity, and wellbeing; and (iii), Rangatiratanga—Taking action; making changes. Collectively, these three sub-themes reflect teina perspectives about “feeling more confident” and viewing the intervention as “self-giving” which relate to them realising their full potential, particularly as kaumātua. They felt the intervention helped them to think differently, with new hope, attitude and understanding. Finally, they viewed the intervention as also helping them take self-determined action because it enhanced their cultural identity and wellbeing. These are hallmarks of mana motuhake.

The second theme centred on whanaungatanga, which refers to connectedness with others, and building, maintaining and enhancing relationships. This theme focuses on whanaungatanga within whānau as well as in terms of wider social connectedness, particularly with other kaumātua. Within whānau, it consisted of strengthened relationships, keeping “in touch” and sharing with whānau what they “had learnt” in the intervention. In terms of wider social connectedness, whanaungatanga centred on strengthened relationships with other kaumātua, reducing loneliness, and getting involved. In summary, this theme highlights both social connectedness and the associated positivity in ageing gained in talking and sharing with others as a result of participating in the intervention.

The third theme focused on tautokotanga, which is about providing assistance or support when needed and may come in many forms including emotional, informational, affirmational, or cultural support. This theme concerned the strengthening of teina knowledge about services or information that could make a difference to them and other kaumātua. The comments demonstrate the ways in which teina experienced tautokotanga—strengthened access to information about “resources” and “services”, as well as “people” who could help. In sum, tautokotanga focused on tangible and information support through services, although that was made possible through increase social connections (i.e., whanaungatanga).

### Cost effectiveness analysis

The second aim was to examine the cost effectiveness of the programme. The comparison of unconditional means (in Table 4) shows a clear (and statistically significant) increase in HRQOL from T1 to T3. However, the ATT shows a small and statistically insignificant effect (coefficient = 2.66; SE = 2.94). The statistical insignificance of the ATT estimates arises primarily because the G2 group experienced a significant increase in HRQOL in the period from T1 to T2, which was before they began to receive the intervention. They then received a twofold further increase in HRQOL between T2 and T3, when they received the intervention, but during that period the G1 group also experienced a further increase in HRQOL (which may have arisen because they continued to receive support and/or services resulting from the intervention). We suspect that the increases in HRQOL that occurred for the groups during the periods when they were not receiving the intervention arise at least partially because of positive spillover effects. Specifically, we have evidence that 73 participants reported talking with other people about the intervention including 46 in G1 and 27 in G2. Nonetheless, we have used the conservative estimate to calculate cost effectiveness.

The total cost of the programme excluding evaluation costs was NZ$258,163. The cost of the status quo (no programme) was assumed to be zero. The coefficient (and standard error) for the cost effectiveness were taken from ATT estimates, as being the full impact of the programme for the *n* = 121 participants who completed the programme. This represents a conservative cost-effectiveness estimate, as 180 participants began the programme. The ICER was estimated 5000 times, using random draws from a normal distribution, based on the coefficient and standard error. The cost per unit increase in HRQOL was NZ$1488 (95% C.I. -$3288, $4054). This can be interpreted as the cost to raise one kaumatua’s HRQOL by one point (on the 0–100 scale). It can also be interpreted as the cost of 0.01 Quality Adjusted Life Years (QALYs), assuming that one QALY corresponds to an increase from 0 to 100 on the HRQOL scale. Scaling this estimate to the cost for one whole QALY results in an estimate of NZ$148,832 (95% C.I. -$322,752, $405,352). Following WHO recommendations, this cost per QALY represents a cost-effective health intervention, as the cost per QALY is less than three times New Zealand GDP per capita (NZ$59,729 for the June 2018 quarter) [70].

However, using GDP per capita as a threshold for assessing cost-effectiveness has been criticised [71]. An alternative threshold could be based on the value of a statistical life year lost (VSLYL), which is estimated at NZ$130,295 (in 2008 NZ$) [69]. Again assuming that this value is equivalent to a 100-point decrease in HRQOL, then this provides suggestive evidence that the intervention is cost-effective, especially noting that our estimate of cost-effectiveness is conservative.

## Discussion

The aim of the study was to examine the impacts of a tuakana/teina peer educator intervention on health and mana motuhake outcomes for kaumātua. A second aim examined the cost effectiveness of the intervention. The analysis revealed improvements over time in the expected direction on most of the variables. However, only three of the variables had statistically significant intervention effects: received support, tribal identity, and trouble paying bills. Qualitative results supported impacts of the intervention on mana motuhake, social connectedness, and tangible/information support related to services. Cost-effectiveness analysis showed that the intervention is cost effective, with a cost per QALY of less than the conventional threshold of three times GDP per capita.

The findings of this study provide further support of the benefit of using peer education with older people. While peer education has been primarily used for younger populations, recent research has found support for its use with older populations in terms of issues such as successful ageing [30], self-management of chronic conditions [31], palliative care [25–27], raising awareness of health [28, 29], and physical activity and fall-prevention [32–35]. The current study illustrates that peer education has effects on specific health and social outcomes for an elder Indigenous community, which is a new contribution to the research literature. The qualitative responses appear to attribute significant impact of the intervention, particularly in terms of social connections, self-efficacy, and informational support for health and social services, which likely contributed to the significant improvement on trouble paying bills.

In addition to being effective, the benefits are also cost effective. The cost per QALY compares favourably with thresholds based on three times GDP per capita and value of a statistical life year lost. In a public health care system like New Zealand, where resources are limited, allocation of funding on the basis of cost-effectiveness is a “moral imperative” [72], and our results strongly support the funding of this intervention within this population.

The study demonstrates that a culturally appropriate peer education intervention contributes to age-friendly social environments that can address social connectedness through cultural concepts such as tribal identity, tautoko, and whakawhanaungatanga [15, 37]. The contributions to social connections are significant given research that finds greater social isolation for Māori relative to other New Zealanders [73], which is particularly important given its strong links to poor health [73, 74].

A further key implication of this study is the importance of grounding the intervention in mātauranga Māori, in particular kaumātua mana motuhake. This intervention introduced a Kaupapa Māori approach that is strengths-based rather than deficiency focussed; it addresses a desire to value older people in all settings. In alignment with mana motuhake, the project utilised Māori culture itself for answers to challenges around life transitions by focusing on and valuing Māori epistemologies surrounding ageing [16, 75]. In particular, the qualitative research illustrated kaumātua feelings of mana motuhake as a result of the intervention.

Additionally, the intervention was kaumātua-led and developed and evaluated through a participatory research approach with a kaumātua-focused organisation. CBPR is a frequently used approached to work with Indigenous communities and to address health inequities [42, 76]. This study provides further evidence of the benefit of CBPR approaches and also demonstrates the successful integration of CBPR and Kaupapa Māori research approaches.

The study is not without limitations. First, the measures in the study were all self-report so direct improvement to health cannot be determined. Nonetheless, wellbeing is subjective and thus appropriate for this study. Further, a couple of the measures had lower than desirable measures of internal consistency. Second, while we attempted to use randomization throughout the selection and assignment process, we were not able to complete full random assignment. Despite the fact that there no differences between those randomly assigned and those not randomly assigned, this does provide a limitation to the research. We chose to be inclusive of participants consistent with Kaupapa Māori philosophy rather than force a Western research expectation. Third, the research design was innovative in that it provided a comparison group without withholding the intervention. However, there were limited intervention effects to fully support the efficacy of the intervention. The presence of positive spillover effects from two intervention groups likely results in ATTs that underestimates the true effect of the intervention. The change in measures over time, coupled with the strong qualitative data, leads us to conclude the intervention was effective and cost-effective, but there is need for further testing to confirm the effectiveness and cost-effectiveness of the intervention in other settings. Future research could address this by utilising a research design that allows for clearer separation of intervention groups, or that specifically seeks to estimate the size of positive spillover effects. An additional future direction is to further target the participants in need of the intervention. We did not use inclusion criteria around needs (e.g., loneliness) and it may be that this intervention is more impactful for those with greatest health and social needs.

## Conclusions

This study resulted in a culturally-safe and effective tuakana/teina peer education intervention to assist kaumātua work through life transitions, resulting in some positive health and social outcomes, particularly related to social connectedness. The intervention is cost effective and has the potential to help a variety of communities help address equity issues in ageing for Māori and potentially other Indigenous and non-Indigenous communities. The positive outcomes in this project can be attributed to the focus on mana motuhake, being kaumātua-led, and being developed through a participatory approach with a community organisation. Participatory research approaches like those used in this study centre local mātauranga and context.

The findings provide evidence for the importance and relevancy of this study’s approach for creating contextually-based and culturally-safe, age-friendly environments that facilitate engagement and participation by kaumātua. From a policy and practice perspective, this intervention has the potential to effectively address key health and social outcomes in a cost effective manner. The intervention may be a viable alternative in the Aotearoa public health system to current practice whereby kaumātua help other kaumatua to address their needs in later-stage life transitions that may help to address some of the health inequities related to aging for Māori. This is also important given the importance of treaty obligations made by the government with Māori to ensure participation, protection and partnership for Māori. The current intervention supports these obligations by taking a strengths-based approach where kaumātua capacity and cultural knowledge instead of a deficit-based approach.

## Data Availability

The datasets used and/or analysed during the current study are available from the corresponding author on reasonable request.

## Abbreviations

CBPR: Community-based participatory research
GDP: Gross domestic product
HRQOL: Health-related quality of life
ICEA: Incremental cost effectiveness analysis
ICER: Incremental cost-effectiveness ratio
QALY: Quality-adjusted life year
RKCT: Rauawaawa Kaumātua Charitable Trust
G1: 1st group
G2: 2nd group

## References

[1] Hayman KJ, Kerse N, Dyall L, Kepa M, Teh R, Wham C (2012). Life and living in advanced age: A cohort study in New Zealand -Te Puawaitanga o Nga Tapuwae Kia Ora Tonu, LiLACS NZ. Study protocol BMC Geriatr.

[2] Blakely T, Shilpi A, Bridget R, Martin T, Martin B (2004). Decades of disparity: widening ethnic mortality gaps from 1980 to 1999. New Zea Med J.

[3] Howden-Chapman P, Blakely T, Blaiklock AJ, Kiro C (2000). Closing the health gap. New Zeal Med J.

[4] Ministry of Health and University of Otago (2006). Decades of disparity III: ethnic and socioeconomic inequalities in mortality, New Zealand 1981–1999.

[5] Rohr M, Lang F (2009). Aging well together: A mini-review. Gerontol..

[6] Kendig H, Browning C, Thomas S, Wells Y (2014). Health, lifestyle, and gender influences on aging well: an Australian longitudinal analysis to guide health promotion. Front Public Health.

[7] Fowler C, Gasiorek J, Giles H (2015). The role of communication in aging well: introducing the communicative ecology model of successful aging. Commun Monogr.

[8] Oetzel JG, Simpson M, Berryman K, Iti T, Reddy R (2015). Managing communication tensions and challenges during the end-of-life journey: perspectives of Māori kaumātua and their whānau. Health Commun.

[9] Oetzel JG, Simpson M, Berryman K, Reddy R (2015). Differences in ideal communication behaviours during end-of-life care for Māori carers/patients and palliative care workers. Palliat Med.

[10] Maclennan B, Wyeth E, Hokowhitu B, Wilson S. Derrett S. Injury severity and 3-month outcomes among Māori: results from a New Zealand prospective cohort study. New Zeal Med J. 2013; 126(1379):39–49. 24045351

[11] Wyeth Emma H, Derrett Sarah, Hokowhitu Brendan, Samaranayaka Ari (2013). Indigenous injury outcomes: life satisfaction among injured Maori in New Zealand three months after injury. Health and Quality of Life Outcomes.

[12] Wham C, Teh R, Moyes S, Dyall L, Kēpa M, Hayman K, Kerse N (2015). Health and social factors associated with nutrition risk: results from life and living in advanced age: a cohort study in New Zealand (LiLACS NZ). J Nutr Health Aging.

[13] Dyall L, Kepa M, Teh R, Mules R, Moyes SA, Wham C (2014). Cultural and social factors and quality of life of Maori in advanced age. Te puawaitanga o nga tapuwae kia ora tonu - life and living in advanced age: a cohort study in New Zealand (LiLACS NZ). New Zeal Med J.

[14] Durie M (1999). Kaumatuatanga: reciprocity: Maori elderly and whanau. New Zeal J Psych.

[15] Mead H (2003). Tikanga Māori: Living by Māori Values.

[16] Hokowhitu B, Kermoal N, Andersen C, Reilly M, Rewi P. Petersen A. Indigenous identity and resistance: researching the diversity of knowledge. Dunedin: University of Otago Press; 2010.

[17] Ajzen I, Fishbein M (1980). Understanding attitudes and predicting social behavior.

[18] Rogers E (2003). Diffusion of innovations.

[19] Bandura A (1986). Social foundations of thought and action: A social cognitive theory.

[20] Braun KL, Kagawa-Singer M, Holden AEC, Burhansstipanov L (2012). Cancer patient navigator tasks across the cancer care continuum. J Health Care Poor Underserved.

[21] Oman RF, Vesely S, Aspy CB, McLeroy KR, Rodine S, Marshall L (2004). The potential protective effects of youth assets on adolescents' alcohol and drug use. Am J Public Health.

[22] Gottfredson DC, Wilson DB (2003). Characteristics of effective school-based substance abuse prevention. Prev Sci.

[23] Karwalajtys T, McDonough B, Hall H, Guirguis-Younger M, Chambers LW, Kaczorowski J (2009). Development of the volunteer peer educator role in a community cardiovascular health awareness program (CHAP): a process evaluation in two communities. J Community Health.

[24] Dennis C-L (2003). Peer support within a health care context: a concept analysis. Int J Nurs Stud.

[25] Goodman C, Iliffe S, Manthorpe J, Gage H, Barclay S, Mathie E (2011). Talking about living and dying with the oldest old: public involvement in a study on end of life care in care homes. BMC Palliat Care.

[26] Lorig K, Hurwicz M, Sobel D, Hobbs M, Ritter P (2005). A national dissemination of an evidence-based self-management program: a process evaluation study. Patient Educ Couns.

[27] Seymour JE, Almack K, Kennedy S, Froggatt K (2013). Peer education for advance care planning: volunteers’ perspectives on training and community engagement activities. Health Expect.

[28] Layne JE, Sampson SE, Mallio CJ, Hibberd PL, Griffith JL, Krupa DS (2008). Successful dissemination of a community-based strength training program for older adults by peer and professional leaders: the people exercising program. J Am Geriatr Soc.

[29] Uitewaal P, Bruijnzeels M, de Hoop T, Hoes A, Thomas S (2004). Feasibility of diabetes peer education for Turkish type 2 diabetes patients in Dutch general practice. Patient Educ Couns.

[30] Kocken P, Voorham A (1998). Effects of a peer-led senior health education program. Patient Educ Couns.

[31] Philis-Tsimikas A, Fortmann A, Lleva-Ocana L, Walker C, Gallo LC (2011). Peer-led diabetes education programs in high-risk Mexican Americans. Diabetes Care.

[32] Khong L, Farringdon F. Hill K, Hill A. "we are all one together": peer educators' views about falls prevention education for community-dwelling older adults - a qualitative study. BMC Geriatr 2015; 15:28. 10.1186/s12877-015-0030-3 PMC4374404 25887213

[33] Little G (2012). Nordic walking for health in Wales: an innovative and successful active ageing programme for older people. J Aging Phys Act.

[34] Stevens Z, Barlow C (2015). Lliffe S. promoting physical activity among older people in primary care using peer mentors. Prim Health Care Res Dev.

[35] Werner D, Teufel J, Brown SL (2014). Evaluation of a peer-led, low-intensity physical activity program for older adults. Am J Health Educ.

[36] Durie M (1998). Whaiora: Māori health development.

[37] Winitana M (2012). Remembering the deeds of Māui: what messages are in the tuakana-teina pedagogy for tertiary educators?. MAI Review.

[38] Tate H (2010). Towards some foundations of a systematic Māori theology: he tirohanga anganui ki ētahi kaupapa hōhonu mō te whakapono Māori.

[39] Oetzel J, Hokowhitu B, Simpson M, Reddy R, Cameron M, Meha P (2019). Kaumātua Mana Motuhake: A study protocol for a peer education intervention to help Māori elders work through later-stage life transitions. BMC Geriatr.

[40] Tuhiwai-Smith L (1999). Decolonizing methodologies: research and indigenous peoples.

[41] Smith GH (1997). The development of kaupapa Māori: theory and praxis.

[42] Wallerstein N, Duran B, Oetzel J, Minkler M (2018). Community-based participatory research for health: advancing social and health equity.

[43] Oetzel J, Villegas M, Zenone H, White Hat E, Wallerstein N, Duran B (2015). Enhancing stewardship of community-engaged research through governance. Am J Public Health.

[44] Kennedy V, Cram F (2010). Ethics of researching with whānau collectives. MAI Review.

[45] Mane J (2009). Kaupapa Māori: A community approach. MAI Review.

[46] Durie M (2005). Ngā tai matatū: tides of Māori endurance.

[47] Walker H. Kai te ao marama. Kai ro pouri tonu ranei: how enlightened are we? Wellington. New Zealand: Victoria University; n.d.

[48] Simpson M, Greensill H, Nock S, Meha P, Harding T, Shelford P, et al. Kaumātua mana motuhake in action: developing a culture-centred peer support programme for managing transitions in later life. Ageing Soc 2019;online:1–24.

[49] Sanders C, Seymour J, Clarke A, Gott M, Welton M (2006). Development of a peer education programme for advance end-of-life care planning. International J Palliat Nurs.

[50] Clarke A, Sanders C, Seymour J, Gott M, Welton M (2009). Evaluating a peer education programme for advance end-of-life care planning for older adults: the peer educators' perspective. Int J Disabil Hum Dev.

[51] Patsopoulos NA (2011). A pragmatic view on pragmatic trials. Dialogues Clin Neurosc.

[52] Oetzel J, Hokowhitu B, Simpson M, Reddy R, Nock S, Greensill H (2019). Correlates of health-related quality of life for Māori elders involved in a peer education intervention. J Health Commun.

[53] Achat HM, Thomas P, Close GR, Moerkerken LR, Harris MF (2010). General health care service utilisation: where, when and by whom in a socioeconomically disadvantaged population. Aust J Prim Health.

[54] Dulin PL, Stephens C, Alpass F, Hill RD, Stevenson B (2011). The impact of socio-contextual, physical and lifestyle variables on measures of physical and psychological wellbeing among Māori and non-Māori: the New Zealand health. Work and Retirement Study Ageing Soc.

[55] Wu A, Revicki D, Jacobsen D, Malitz F (1997). Evidence for reliability, validity and usefulness of the medical outcomes study HIV health survey (MOS-HIV). Qual Life Res.

[56] Statistics New Zealand. Te Kupenga 2013: A survey of Māori well-being 2013.

[57] Unger J, McAvay G, Bruce ML (1999). Variation in the impact of social network characteristics on physical functioning in elderly persons: MacArthur studies of successful aging. J Gerontol B Psychol Sci Soc Sci.

[58] Oeki M, Mogami T, Hagino H (2012). Self-perceived burden in patients with cancer: scale development and descriptive study. Eur J Oncol Nurs.

[59] Kroemeke A (2015). Perceived autonomy in old age scale: factor structure and psychometric properties of the polish adaptation. Psychiatr Pol.

[60] Cantril H (1965). The pattern of human concerns.

[61] Hussey M (2007). Hughes. Design and analysis of stepped wedge cluster randomized trials Contemp Clin Trials.

[62] Nickless A, Voysey M, Geddes J, Yu L-M, Fanshawe T (2018). Mixed effects approach to the analysis of the stepped wedge clsuter randomized trial--investigating the confounding effect of time through simulation. PLoS One.

[63] Braun V, Clarke V (2006). Using thematic analysis in psychology. Qual Res Psychol.

[64] Patton MQ (2002). Qualitative research and evaluation methods.

[65] Owen W (1984). Interpretive themes in relational communication. Q J Speech.

[66] Gold MR, Siegel JE, Russell LB, Weinstein MC (1996). Cost-effectiveness in health and medicine.

[67] Edejer T, Baltussen R, Adam T, Hutubessy R, Acharya A, Evans D (2003). Making choices in health: WHO guide to cost-effectiveness analysis.

[68] Sindelar J, Jofre-Bonet M, French M, McLellan A (2004). Cost effectiveness analysis of addiction treatment: paradoxes of multiple outcomes. Drug Alcohol Depen.

[69] O'Dea D, Wren J (2010). New Zealand estimates of the total social and economic cost of “all injuries” and the six priority areas respectively, at June 2008 prices: technical report prepared fro NZIPS evaluation.

[70] Statistics New Zealand. Gross domestic product: 2018. Wellington, NZL: Statistics New Zealand. Retrieved from https://www.stats.govt.nz/information-releases/gross-domestic-product-june-2018-quarter.

[71] Robinson L, Hammitt J, Chang A, Resch S (2017). Understanding and improving the one and three times GDP per capita cost-effectiveness thresholds. Health Policy Plann.

[72] Ord T (2013). The moral imperative toward cost-effectiveness in global health.

[73] Wright-St. Clair V, Neville S, Forsyth V. Integrative review of older adult loneliness and social isolation in Aotearoa/New Zealand. Aust J Ageing 2017;36:114–123. 10.1111/ajag.12379 PMC5484290 28258607

[74] Chuang Y-C, Chuang K-Y, Yang T-H (2013). Social cohesion matters in health. Int J Equity Health.

[75] Hokowhitu B (2009). Indigenous existentialism and the body. Cult Stud Rev.

[76] Moffitt Pertice, Dickinson Raissa (2016). Creating exclusive breastfeeding knowledge translation tools with First Nations mothers in Northwest Territories, Canada. International Journal of Circumpolar Health.

